# Rapid molecular test (SeptiFast^®^) reduced time for adjustment of antibiotic treatment in comparison with conventional blood cultures in critically ill sepsis patients: a randomized controlled clinical trial (preliminary results)

**DOI:** 10.1186/cc12926

**Published:** 2013-11-05

**Authors:** Cristhieni Rodrigues, Mirlane Silva dos Santos, Helio Hehl Caiaffa Filho, Cecilia Eugênia Charbel, Luciane de Carvalho Sarahyba da Silva, Flávia Rossi, Maria Renata Gomes Franco, Rinaldo Focaccia Siciliano, Tânia Mara Varejão Strabelli

**Affiliations:** 1 Infection Control Unit, Heart Institute (InCor), University of Sao Paulo Medical School, Sao Paulo, Brazil; 2 Molecular Biology Branch, Central Laboratory Division, University of Sao Paulo Medical School, Sao Paulo, Brazil; 3 Central Laboratory Division, University of Sao Paulo Medical School, Sao Paulo, Brazil

## Background

Sepsis is the main cause of death in ICUs all over the world. Early detection of the pathogen is essential for appropriate antimicrobial treatment.

## Materials and methods

To evaluate the reduction in time of antimicrobial adjustment therapy in patients with sepsis comparing a rapid molecular test (SeptiFast^®^) with conventional blood cultures, a randomized controlled clinical trial was conducted between October 2012 and May 2013 in a cardiology hospital. Adult patients staying more than 48 hours in hospital with clinical suspicion of sepsis were included in the study. Blood samples were collected for cultures (BacT/ALERT^®^) and Septifast^® ^test immediately prior to initiation of antibiotic therapy. Patients were allocated into two groups. In the Intervention Group (GI), Septifast^® ^results were communicated to the medical researcher and antimicrobials were adjusted. In the Control Group (GII), Septifast^® ^results were not informed and therapy adjustment was based on the blood culture. Registered in Clinical trials.gov (NCT 01450358).

## Results

Forty-six patients were included, 17 in GI and 29 in GII. Key data are shown in Table 1. In GI therapy adjustment was done in 580 minutes compared with 3,007 minutes in GII (*P *= 0.004).

**Table 1:** Distribution of characteristics in the two groups

	Intervention group (*n*= 17)	Control group (*n*= 29)	*P *value
Age (years)	63 (46 to 75)	66 (39 to 85)	0.340
Gender (female)	5 (30%)	10 (34%)	0.999
Hospital stay (mean, days)	32 (9 to 118)	31 (3 to 112)	0.632
APACHE II (mean)	17 (8 to 29)	17 (8 to 29)	0.730
Ejection fraction <40%	8 (47%)	17 (58%)	0.545
Receiving antibiotics prior to study	11 (65%)	11 (38%)	0.126
Septic shock	9 (53%)	16 (55%)	0.999
Patients with adjustment therapy based on SeptiFast^®^	6 (35%)	-	
Patients with adjustment therapy based on blood culture	-	7 (21%)	
Mean time (minutes) of adjustment therapy	580	3,007	0.004
Pathogens detected in SeptiFast^®^	*P. aeruginosa *(2), *K. pneumonia/oxytoca *(1), *E. aerogenes/cloacae *(1), *S. marcescens *(1), *A. baumanii *(1)	-	
Pathogens detected in blood culture	-	*S. aureus *(4), *K. pneumonia *(2), *M. morganii *(1)	
28-day mortality	9 (53%)	17 (59%)	0.765

## Conclusions

The rapid molecular test (SeptiFast^®^) reduced the time for adjustment of antibiotic treatment in comparison with conventional blood cultures in critically ill sepsis patients.

